# Backward reasoning through AND/OR trees to solve problems

**Published:** 2024

**Authors:** Jeroen Olieslagers, Zahy Bnaya, Yichen Li, Wei Ji Ma

**Affiliations:** Center for Neural Science, New York University, New York, NY, United States; myWhatIf foundation, Tel Aviv, Israel; Department of Psychology, Harvard University, Cambridge, MA, United States; Center for Neural Science and Department of Psychology, New York University, New York, NY, United States

**Keywords:** problem solving, backward reasoning, subgoals, AND/OR trees, Rush Hour

## Abstract

Whether travelling, playing games, or debugging code, any situation where an agent desires change can be framed as a problem. Despite this ubiquity, there is no unifying framework describing how people reason backwards when solving problems. We introduce AND/OR trees, which chain together subgoals and actions to attain them, as a way to represent this process. To investigate whether actions from AND/OR trees were predictive of human behavior, we conducted a study in which participants solved deterministic, long-horizon puzzles. AND/OR trees were able to explain most of the actions the participants took. Next, we modeled search through these trees using a psychologically plausible, single-parameter search algorithm. We fit this model to the data of individual participants and found that it captures trends in summary statistics of human play. Our results show the promise of AND/OR trees as a representation for backward reasoning in problem solving.

## Introduction

The field of human problem solving, popularized by Newell and Simon (1972), has a long and distinguished history in the field of cognitive science. One of the central pillars of human problem solving is search (Simon, 1983; Wang & Chiew, 2010). This process can happen forward, as is commonly studied in the planning literature (Callaway et al., 2022; Correa, Ho, Callaway, Daw, & Griffiths, 2023; Russek, Acosta-Kane, van Opheusden, Mattar, & Griffiths, 2022; van Opheusden et al., 2023), or backward, as is commonly studied (in tandem with forward search) in the problem solving literature (Choi, Kaufman, Langley, Nejati, & Shapiro, 2004; Kobylarz, DeBar, Reeve, & Meyer, 2020; Larkin, McDermott, Simon, & Simon, 1980; Park, Lu, & Hedgcock, 2017). However, these studies focused on either developing a broader, phenomenological problem solving framework or investigating in which situations people reason backwards, and not on creating a parameterized process model fit on human data to predict behavior.

The field of automated planing has a long history of using and contributing to theories of human problem solving (Fikes & Nilsson, 1971; Newell, Shaw, & Simon, 1959; Shapiro, 1979). However, many modern day automatic problem solvers have shifted away from modeling human problem solving in order to better solve problems. Instead, they are designed to push the frontier of what problems computers are capable of solving, without much regard to human cognition (Georgievski & Aiello, 2016; Nau, Cao, Lotem, & Munoz-Avila, 2001; Raad et al., 2024; Trinh, Wu, Le, He, & Luong, 2024).

## Backward reasoning

If you are presented with a model of the world (transition probabilities in the framework of Markov Decision Processes), but little information about where exactly you wish to end up, then forward search is an efficient method to mentally explore the state space until you find a desirable state. However, in forward search, actions that have no connection to the goal state(s) might be considered, which can be a waste of computation.

If you do have information about the goal state: where you wish to end up, then you should leverage that to make search easier. This is what backward search aims to address. In backward search, every action considered is linked to reaching the goal state. This is because the process starts at the goal and works backward to where the agent is. If this process is done in the state space of the problem, then the agent might consider states that are unreachable, also wasting computation.

We propose that backward reasoning (distinct from backward search), can mitigate this inefficiency in backward search. Backward reasoning is similar to backward search, but instead of a search over states, it is a search over (sub)goals. The agent starts at the goal, and breaks it down into subgoals, which themselves get broken down into subgoals. This process continues until the problem is sufficiently subdivided. From this point on, the agent selects one of the subgoals, and either immediately attains it, or performs forward/backward search to find an action to attain it. We contribute to the field of human problem solving by providing a representation to explain the human thought process during backward reasoning.

To describe the process of backward reasoning in problem solving, imagine that you are hungry. Solving this problem involves eating something, which becomes your goal. To attain this goal, you may consider eating a salad, burger, or lasagna. If you decide on lasagna, then the new problem is that it is not immediately available to you, which creates a subgoal: in order to eat lasagna, you must first cook it. To accomplish this subgoal, you could heat up store-bought microwave lasagna, or you could make lasagna from scratch in the oven or air fryer. Since you already have all the ingredients, you decide to cook lasagna in the oven. The action of putting lasagna in the oven and cooking it is not available to you. This time, multiple subgoals present themselves: you must preheat the oven, prepare the sauce and boil the pasta. Only after accomplishing all these subgoals may you proceed to cook the lasagna in the oven. Going down this train of thought, you decide that the first action you will take to solve the problem of sating your hunger is to preheat the oven.

## AND/OR trees

We propose that the process of chaining subgoals together during backward reasoning can be represented using AND/OR trees. AND/OR trees were first used by Slagle to automatically solve analytical integration problems (Slagle, 1963). They were then used in other artificial intelligence efforts such as theorem proving (Amarel, 1967; Kowalski, 1972; Vanderbrug, 1976), language recognition (VanderBrug & Minker, 1975), two player games (Pearl, 1984; Slagle & Dixon, 1969), optimizing decision tables (Martelli & Montanari, 1978), and more recently in constrained optimization (Marinescu & Dechter, 2004) as well as solving games (Kishimoto, Winands, Müller, & Saito, 2012).

AND/OR trees are similar to decision trees in that they have a root node that is connected to child nodes by directed edges, ending at leaf nodes. In AND/OR trees, however, there is a distinction between AND nodes and OR nodes. AND nodes are considered ‘satisfied’ if *all* of their child nodes are satisfied. OR nodes are satisfied if *any* child node is satisfied. In the context of problem solving, OR nodes are subgoals and AND nodes are actions that attain subgoals. These are then chained together as follows: a subgoal has as child nodes a set of possible actions that each attain the subgoal. These actions might not immediately be available to the agent (just like eating lasagna isn’t possible if you haven’t cooked it yet), but if any one is taken, it will result in the attainment of the subgoal. If an action *is* immediately available to the agent, it is a leaf node and will have no child nodes. However, if the action is *not* immediately available, a set of subgoals will be created. This can be thought of as asking “What has to be done before I can take this action?”. The actions are AND nodes since *all* subgoals have to be attained before the action becomes available.

The whole process starts at the root node, which is the overall goal of the problem and is hence an OR node. Taking any action that is a child node of the root will result in the problem being considered solved. AND/OR trees provide a useful representation because every action considered contributes to attaining the overall goal that solves the problem.

## Task

To investigate whether people reason backward through AND/OR trees to solve problems, we want a task that (1) has a clear goal; (2) naturally breaks down into subgoals; (3) has a tractable state space and computable optimal solutions; (4) is complex enough to require multiple steps of thinking; (5) has minimal learning, perceptual, social and language components; and (6) has deterministic transitions and is set in a deterministic environment. While we propose AND/OR trees generalize to all tasks that satisfy (1), we wish to focus on investigating the structure of the tree, and avoid any possible confounds through (2–6).

**Figure 1:**
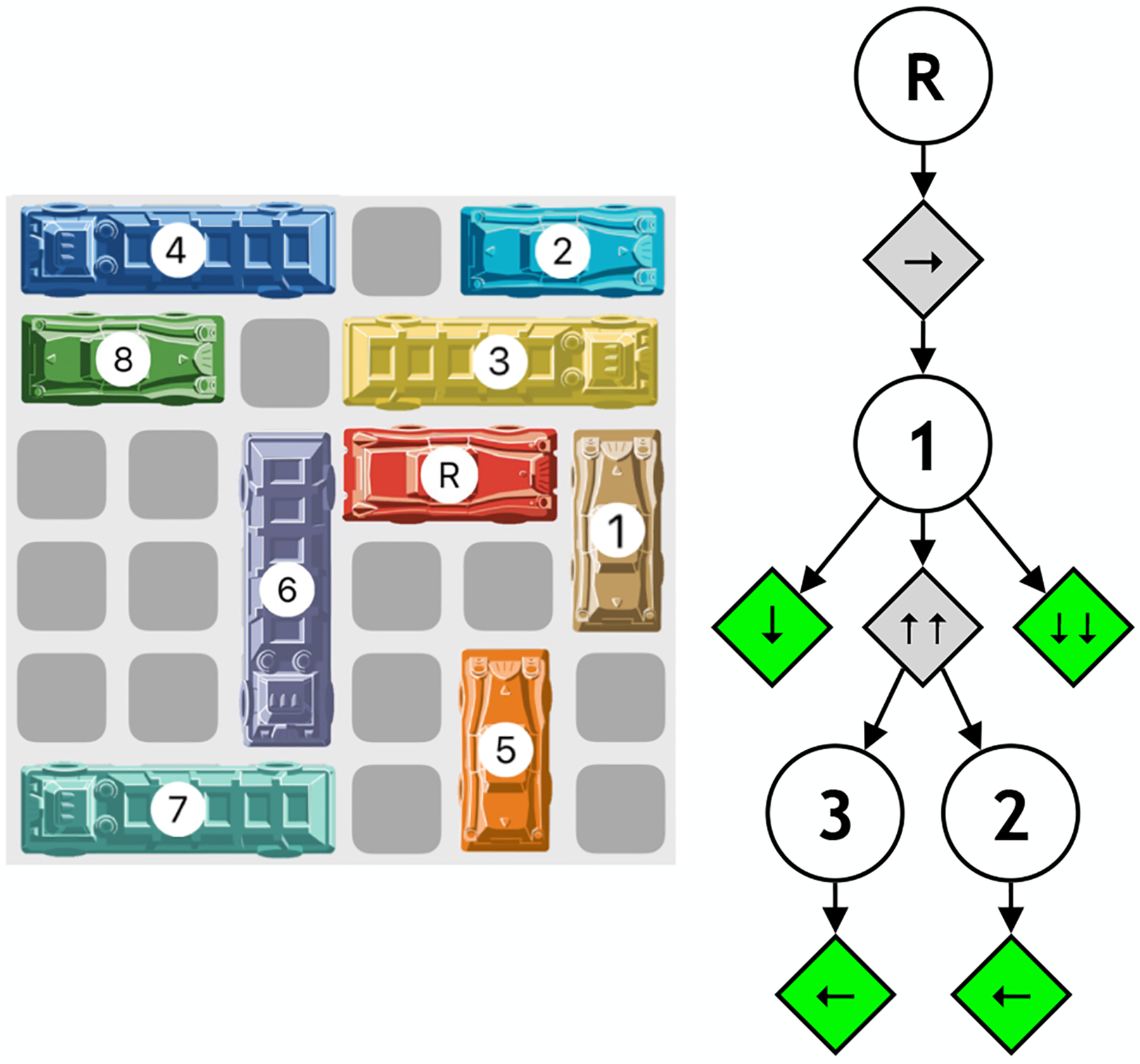
Rush Hour example puzzle (left) with associated AND/OR tree (right). The goal of the puzzle is to move the red car labeled ‘R’ all the way to the right of the board.

All these desiderata are satisfied by the game of Rush Hour: a game created by Nob Yoshigahara in the 1970s and part of the family of “sliding block” puzzles (Spaans, 2009). It is played on a 6 × 6 board where “cars” are pieces occupying either two or three squares, arranged vertically or horizontally (Fig. 1). You may move cars only forward or backward in the direction they are facing. The goal of the game is to move the target car (the red car) out of the board by moving it all the way to the right. The key constraint is that cars may not overlap and you may not move a car through another (or lift it from the board). In the simple example from Fig. 1, the optimal solution (solution requiring the fewest actions) is to move Car 1 down one or two spots, clearing the way for the red car to move all the way to the right. Moving a car any number of spots at once is considered a single action. For this puzzle, the ultimate goal is to move the red car one space to the right. This is depicted in the top (root) node in the AND/OR tree next to the puzzle (each unique board state has its own AND/OR tree). The goal is to free the red car (first OR node) and to do this, it must be moved one spot to the right (first AND node). You will find that this action which solves the puzzle is not immediately available, since Car 1 is blocking the way. Hence, the new and only subgoal that needs to be attained to make this action possible is to move Car 1 out of the way. To do this, you can move Car 1 downwards (one or two spaces) or upwards (two spaces). If you wish to move the car downwards, there is nothing blocking you from doing so, and hence you will have reached a leaf node. Otherwise, if you wished to move Car 1 upwards, you will find that is blocked by both Car 2 and Car 3. In this very simple case, it is clear that moving Car 1 downwards instead of upwards in order to unblock the red car is the better move, but in more complicated puzzles, it is not always so clear. Rush Hour has been studied in the context of determining the complexity of a puzzle (Bennati, Brussow, Ragni, & Konieczny, 2014; Bockholt & Zweig, 2015; Jarušek & Pelánek, 2011) as well as theory of mind (Berke, Tenenbaum, Sterling, & Jara-Ettinger, 2023). However, the field lacks any mechanistic explanation of how people solve Rush Hour puzzles, and to our knowledge there have been no process models of human behavior in this paradigm.

## Experiment

**Figure 2:**
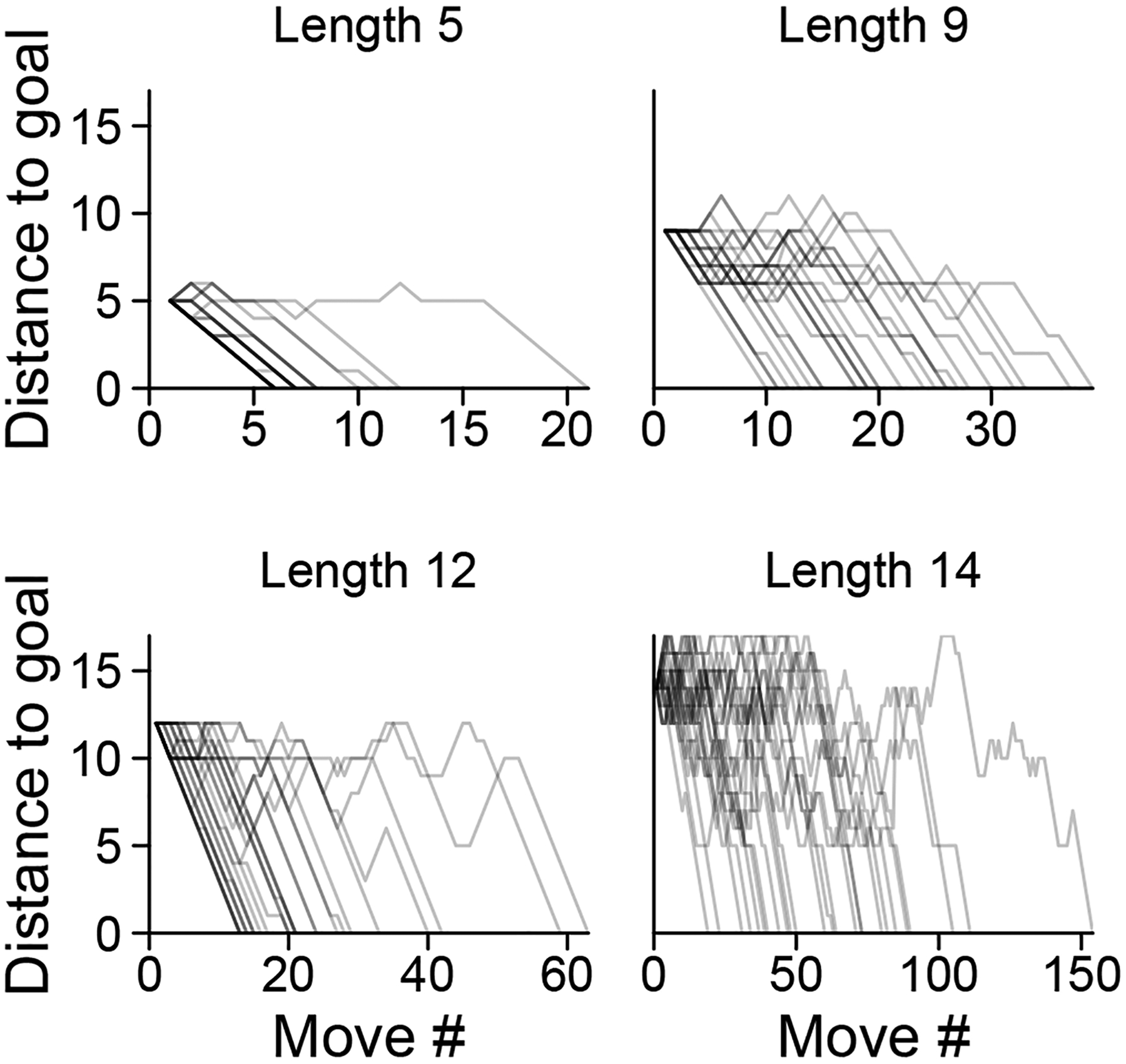
Trajectories from all participants in four randomly selected puzzles, one from each difficulty category. Distance to goal represents the minimum number of actions it takes to reach a goal state, and length is the optimal solution length from the starting state. Darker lines mean more trajectories overlap.

We implemented a web-based version of the game to recruit *n* = 42 participants through Amazon Mechanical Turk. In the experiment, we asked participants to solve any number of 70 Rush Hour puzzles. They could skip puzzles if they found them too hard but received a bonus for completing more puzzles. Puzzles were split into four groups of differing optimal solution length (5, 9, 12, or 14). Every puzzle contained 9 cars. Participants attempted 52 ± 17 (S.D.) puzzles, with a completion rate of 86 ± 14% (S.D.). Across the 42 participants, we obtained a total of 46,965 board states. Analysis of the raw data suggests that participants find it harder to solve puzzles which have a greater optimal solution length (Fig. 2).

## Model

To model how people might be traversing AND/OR trees, we assumed a person stops at any given OR node with probability *γ*. This parameter is directly related to how deep into the tree a participant explores. It also aims to incorporate factors such as attention, motivation, and working memory capacity, without explicitly representing these in the model. The motivation for having a stopping probability is the assumption that people prefer shorter trains of thought compared to longer ones. From a resource-rational point of view (Lieder & Griffiths, 2020), this makes sense: to save on memory and computation, people should prefer to solve the puzzle in as few actions as possible (even if they don’t have this as an explicit goal). A stopping probability like this can also be found in planning algorithms where it serves to limit the depth of planning (van Opheusden et al., 2023).

When a person comes to a point where an AND node requires multiple subgoals to be attained, we assume that they form a probability distribution over these subgoals, where the probability of choosing any one subgoal depends on the heuristic function h^A^, the properties of the subgoal in question OR_*i*_, and the parameters *θ*^A^. These three factors allow us to represent heuristic functions people might use to prefer one subgoal over another (e.g. one subgoal might on the surface appear easier, making it more or less appealing). Similarly, for choosing an action to attain a subgoal, we assume a heuristic function h^O^, the properties of the action in question AND_*i*_, and the parameters *θ*^O^ influence the probability a person chooses one action over another to attain a specific subgoal (e.g. people might select an action that moves a car fewer spaces over an action that moves a car more spaces). These two types of heuristics likely play a significant role in how people arrive at their chosen action. However, since the space of possible heuristic functions is infinite, we started by investigating the simplest case where the heuristic functions are uniform over all options. This means that the only parameter in our model is the stopping parameter *γ* which we fit on a per-participant basis. We experimented with other parameters such as a lapse rate as well as more problem specific parameters but found that these did not provide sufficient improvement to warrant separate investigation.

Since the AND/OR trees we are dealing with are fairly small (none have more than a few hundred nodes, and most have on the order of tens), we don’t have to simulate trajectories through the AND/OR tree as is common in forward search planning algorithms (van Opheusden, Acerbi, & Ma, 2020). We can find the probability of reaching each leaf node by starting at the root node, propagating probabilities downward by breadth-first search. If a cycle were about to form, we terminate the process and the resulting propagated probability gets spread proportionally over all other leaf nodes. Behaviourally, this is equivalent to reaching a cyclic train of thought and starting over.

**Figure 3:**
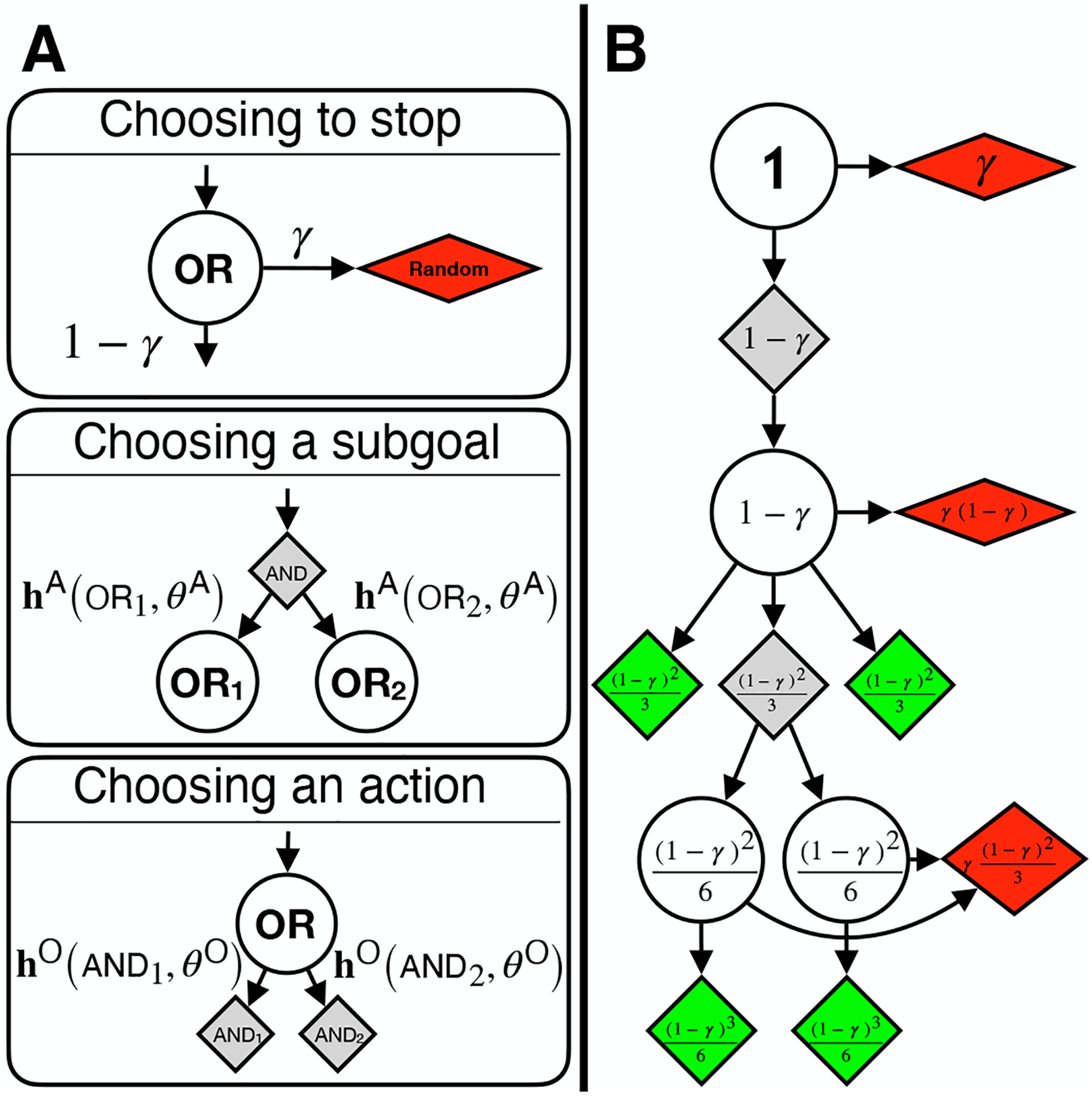
**A.** Probability propagation rules. **Top:** stopping probability *γ*. **Middle:** choosing which subgoal to pursue using heuristics. **Bottom:** choosing which action to take to attain the subgoal using heuristics. **B.** Probability propagation for example puzzle from Fig. 1 where heuristics are uniform over all options.

The propagation process (Fig. 3) works as follows: at the root node, we start with full probability. Since the root is an OR node, we assume people stop with probability *γ* and take a random action. This would be considered a lapse, where the person simply took a random action without thinking about the problem at all. With probability 1 − *γ*, the person continues down the AND/OR tree, looking at which actions attain the overall goal of moving the red car out of the board. They then reach the next subgoal (which in this example is moving Car 1 out of the way of the red car, Fig. 1), where they again stop with probability *γ*. Since they will reach this node with probability 1 − *γ*, the resulting probability of stopping at this node is *γ*(1 − *γ*), and the probability of moving on is (1 − *γ*)^2^. Since there are three ways to move Car 1 out of the way of the red car, and since we have assumed a uniform heuristic function, each child AND node gets a third of the probability: $\frac{{\left(1-\gamma \right)}^{2}}{3}$. This process continues until all nodes in the AND/OR tree have been visited. The result of the process is a probability distribution over actions, where the probabilities of the green nodes get assigned to their respective action, and the summed probability from the red ‘random’ nodes gets spread over all possible actions (as if picking an action at random). We call the green nodes (the AND/OR tree leaves) the sensible actions of the current board state, since each of these actions attains a subgoal that is connected to solving the main goal of the problem. See Fig. 1 for an example: try and make an argument for any of the actions not in the AND/OR tree.

We found the value of *γ* for a given participant by maximum likelihood estimation. Before any optimization, we set *γ* = 0 and noted the resulting probabilities as well as depths of the leaf nodes. Subsequently, we defined a likelihood function which takes in a value for *γ* (between 0 and 1), and multiplies the probability of each leaf node by ${\left(1-\gamma \right)}^{d_{\text{tree}}}$ where *d*_tree_ is the depth of the leaf node in the AND/OR tree. The probability of taking a random action was then calculated as one minus the sum of the probabilities in all the leaf nodes. This allowed us to quickly calculate the likelihood of any action without needing to propagate the probabilities throughout the AND/OR tree over and over. This was only possible since we were not optimizing any heuristic function parameters. The log-likelihoods of all actions from a given participant were summed and maximized using Brent’s method. By plotting the log-likelihood for a fine grid of values of *γ* between 0 and 1, we confirmed that the objective surface was smooth, and that there was a unique maximum for each participant (mean = 0.07, S.D. = 0.025).

To validate this model of backward reasoning, we compared performance against a baseline model called the Eureka model. This model aims to capture the trend seen in the data in Fig. 2 that participants appear to behave randomly up to a point where they seem to have gained some insight (the eureka moment). Similarly to Jarušek and Pelánek (2011), the model assumes that if the person is more than *d* steps away from the nearest goal state, they are unable to see deep enough into the forward search tree to find a goal state. When this happens, the person is assumed to act randomly. If they are *d* steps or fewer away from the nearest goal state, they are assumed to have found a goal state by looking into the decision tree far enough. In these states, they act optimally with probability 1 − *λ*, otherwise they act randomly. This is to model any lapses that might occur. These two parameters are fitted on a per-participant basis as before.

## Results

**Figure 4:**
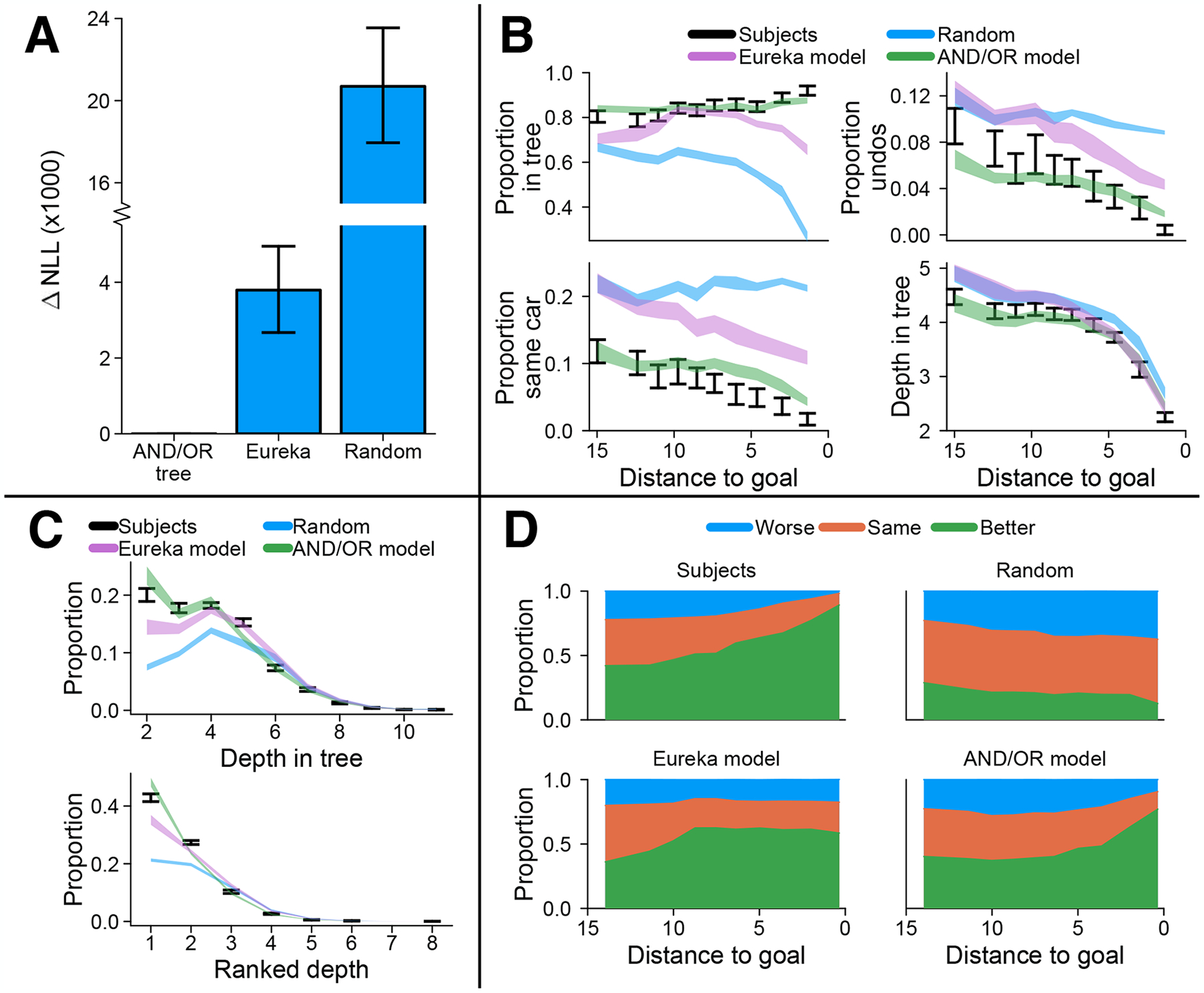
**A.** 5-fold cross-validated negative log-likelihood across *N* = 42 subjects. Errorbars represent 95% confidence intervals. **B.** Summary statistics for each quantile binned distance to goal. *Top left*: proportion of actions that were part of the AND/OR tree. *Top right*: proportion of actions that undid the previous move. *Bottom left*: proportion of actions that moved the same car as previous move. *Bottom right*: average depth of actions which were part of the AND/OR tree (actions that were not part of the tree were excluded). Error bars and shaded areas are 95% confidence intervals around the mean across subjects. **C.** Histograms of actions by depth in AND/OR tree (top) and ranked depth (bottom). Error bars and shaded areas are 95% confidence intervals around the mean across subjects. **D.** Proportion of ‘worse’ (push agent farther from goal), ‘same’ (leave agent at same distance from goal) and ‘better’ (push agent closer to goal) actions for each quantile binned distance to goal. Random agent shows the proportions available to participants and models.

To measure the strength of our AND/OR tree model, we look at its 5-fold cross-validated negative log-likelihood (NLL) relative to the Eureka baseline model. We found a significant improvement, *t*(41) = 90.7, *p* = < .001 (Fig. 4A). From this we can conclude that even though the Eureka model has more parameters, it does a poorer job of predicting participants’ moves. The other problem with the Eureka model is that it fails to explain how a person would know which move is optimal once they are within *d* steps from the goal.

Since Rush Hour board states are abstract, high-dimensional objects, we use summary statistics to capture key information about states and actions. The key summary statistic describing a state is the minimum number of actions it takes to reach a goal state, called the “Distance to goal”. We calculated this summary statistic for every state by performing breadth-first search starting at the state in question, finishing when a goal state is reached (puzzles usually have multiple goal states). We also investigated other summary statistics describing the board state such as the number of available actions, but found these to be uninformative.

We first looked at the proportion of actions participants took that were part of AND/OR trees (the proportion of sensible actions the participants took). We find that this proportion is high: going from 80% in states far from the goal to 90% in states close to the goal (Fig. 4B). However, if the AND/OR tree included 80–90% of all possible actions into its structure, this would not be a surprising result. In this case, 80–90% of the actions taken by a random agent would be included in the AND/OR tree, suggesting the representation is no more useful than a random selection of actions. In reality, the proportion of all possible actions that are part of the AND/OR tree is much smaller (Fig. 4B, blue area). This shows that actions obtained from reasoning through the AND/OR tree are predictive of human actions (up to some noise ceiling). We also find that the proportion of all possible actions that are part of the AND/OR tree drastically reduces closer to the goal. This means that near goal states, there are few sensible actions, while far from the goal, more actions are sensible since there are more ways to reach a goal state. Our model faithfully captures these trend seen in the data (Fig. 4B, overlapping errorbars).

In Rush Hour, state transitions are bidirectional, meaning that actions can always be undone. An optimal agent would never do this, but humans do. Furthermore, the proportion of undos (moving a car back to where it just came from), is found to decrease as participants get closer to a goal state (Fig. 4B). More generally, we also investigated the proportion of actions where the same car was moved as in the previous action. This includes undos but also other erroneous actions such as moving a car forward one space, and then moving it forward one space again. We again find a decrease in these types of moves the closer participants get to the goal. These findings suggest that people make fewer mistakes the nearer to the solution they get, a hypothesis we further investigated below. Our model captures both of these behaviours.

Next, we wanted to find out how the structure of AND/OR trees changes over the course of solving a puzzle. We found that the farther a state was from a goal state, the farther the AND/OR tree leaf nodes were from the root (measured by the depth in tree). Intuitively, the farther a state is from a goal, the more steps are required to reach that goal, and hence the more subsequent subgoals and actions there are. We found that actions nearest to the root of the AND/OR tree (main goal) were most predictive of actions participants took (Fig. 4B). Our model is also able to replicate these trends. This result confirms our initial motivation for the stopping probability parameter *γ*: participants prefer shorter trains of thought over longer ones. In our model, this is replicated by deeper nodes going through more multiplications of 1 − *γ*, receiving smaller probabilities and becoming less likely. This effect of *γ* induces an exponential distribution over depths in the AND/OR tree. We can see this explicitly in the data by ranking the depths of each leaf node in the tree and plotting a histogram of the ranks of chosen moves by participants (Fig. 4C).

The directionality induced by measuring the distance to goal allowed us to classify actions into three categories: actions that increase this distance by 1 which we call ‘worse’ actions, actions that leave the distance unchanged called ‘same’ actions, and actions that decrease the distance by 1 called ‘better’ actions. The latter of these describes optimal actions, since by definition you cannot get closer to a goal state by more than 1 through any one action. The reverse is true for ‘worse’ actions: you cannot get farther from the goal by more than 1 through any one action. We will refer to the collection of ‘worse’ and ‘same’ actions as mistakes. What we see is exactly what we predicted earlier: the proportion of actions which are mistakes decreases as participants get near goal states (Fig. 4D). Closest to the goal, participants act optimally ~85% of the time and take essentially no ‘worse’ actions. This could suggest that when near to the goal, participants can accurately identify which actions are clearly wrong and avoid them. This is further solidified by evidence that in general, the proportion of actions available to participants that are mistakes *increases* as they get closer to the goal (Fig. 4D, Random). This is likely a property of broader problem solving since nearer goal states, the number of paths (defined as trajectories through a forward-search decision tree) to reach a goal state optimally is necessarily the same or smaller than farther from goal states. The smooth qualitative trend seen in the data is captured by the AND/OR tree model, but not by the Eureka model, which has a sharp discontinuity caused by the sharp cutoff *d* induces.

## Discussion

In this paper, we have introduced and shown the merits of AND/OR trees as a way to represent the human thought process of backward reasoning in problem solving. We have also proposed a simple model for traversing these trees, and demonstrated its ability to recapitulate behaviours seen in humans. We showed that participants prefer actions that arise from shorter trains of thought, a finding supported by resource-rationality (Lieder & Griffiths, 2020). Furthermore, we found that human behaviour is most predictable when they are closest to solving a problem, where the number of (optimal) paths to a goal state is smallest. Finally, we showed that people make fewer mistakes the closer they are to solving a problem.

A challenge in bridging our work to real-world problem solving is that the latter often comes with strong heuristics. For example, in the cooking example heuristics would be used in deciding whether the person wanted to eat lasagna, salad or a burger, and would determine whether the person would choose to first cook the pasta or turn the oven on. In the future, we would like to investigate the role heuristics play in shaping the AND/OR tree search process. This would allow us to ask questions about participant-specific biases and make more accurate predictions about their exact thought process.

As is common in the field of cognitive modeling, we have treated each state independently, making the model fitting process simpler, but ignoring important structure present in across-state correlations. Most importantly, people likely form a train of thought, and then execute multiple actions according to the same train of thought. It is unlikely that people form an entirely new train of thought after every action, and people likely carry over information from the AND/OR tree across states. We would like to model this in future work, making predictions about when people form new trains of thought and validating this with reaction time or eye movement data. Finally, we would like to investigate how AND/OR trees can be combined with forward search algorithms to provide a more holistic account for human behaviour across a wider range of tasks.
